# 1886. Prevalence Of Latent TB Infection Among Critically Ill COVID-19 Population In a Tertiary Teaching Hospital In Mexico

**DOI:** 10.1093/ofid/ofad500.1714

**Published:** 2023-11-27

**Authors:** Santiago Montiel, Benjamin Valente-Acosta

**Affiliations:** The National Institute of Medical Sciences and Nutrition Salvador Zubirán (INCMNSZ), Benito Juárez, Distrito Federal, Mexico; ABC Medical Center, CDMX, Distrito Federal, Mexico

## Abstract

**Background:**

Tuberculosis (TB) is the leading cause of death from a single infectious agent. Latent tuberculosis infection (LTBI) is defined as TB infection without clinical symptoms or microbiological evidence. LTBI is a risk factor to later development of active TB. COVID-19 pandemic represents an emerging infectious disease. Immunosuppressants as corticoids and tocilizumab are the standard of care of critically ill COVID-19 patients. Those drugs have previously been shown to increase the risk of developing active TB, especially in patients with latent TB.

**Figure:**
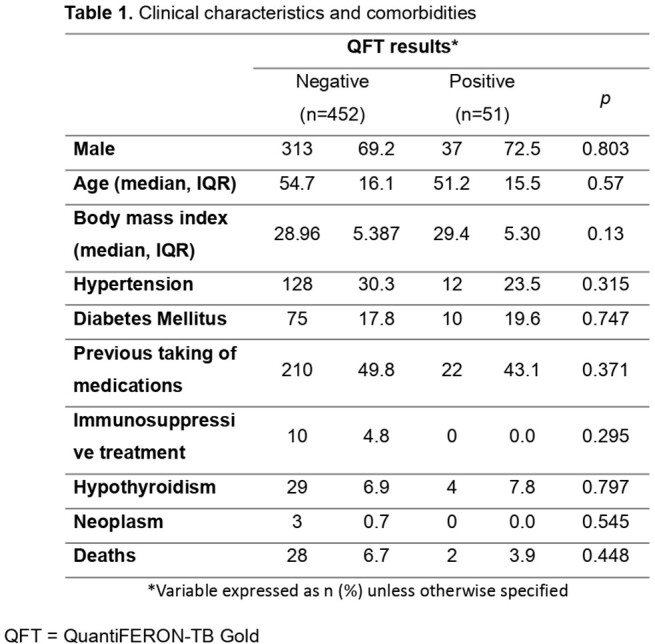

**Methods:**

We conduct a cross-sectional study to determine the prevalence of latent tuberculosis infection, through QuantiFERON, in patients who have been hospitalized with severe or critical COVID-19 at the ABC Medical Center in Mexico City-Mexico, from March 2020 to March 2021.

**Results:**

From 1174 records reviewed, a total of 503 patients who have a QuantiFERON® test were included. The prevalence of LTBI in hospitalized patients with severe or critical COVID-19 in our hospital was 10.1% (n=51). Of the total of patients with a diagnosis of LTBI, 82.3% had severe disease and 17.6% had critical illness requiring invasive mechanical ventilation (IMV) and 42 (82%) received corticosteroids and 29 (57%) received tocilizumab.

**Conclusion:**

To the best of our knowledge this is the first report that depict the LTBI prevalence in a cohort of severe and critical COVID-19 patients in a Lower-Middle Income Country. Our prevalence was lower than previously reported global prevalence of LTBI.

**Disclosures:**

**All Authors**: No reported disclosures

